# Human Infection with G12 Rotaviruses, Germany

**DOI:** 10.3201/eid1509.090497

**Published:** 2009-09

**Authors:** Corinna Pietsch, Uwe G. Liebert

**Affiliations:** Leipzig University, Leipzig, Germany

**Keywords:** rotavirus, gastroenteritis, molecular epidemiology, genotype, genetic variation, diarrhea, enteric diseases, viruses, dispatch

## Abstract

Rotavirus group A G12 genotypes were detected in 3 (1.5%) of 198 stool samples positive for human rotavirus. G12P[6] was present in 2 samples, and a mixed G3G12P[8] was found in 1 sample. Phylogenetic analysis of complete open reading frames of all 11 genomic RNA segments proved their Wa-like genogroup affiliation.

Rotaviruses are worldwide enteric pathogens in humans and animals. Most prevalent human strains in Europe are group A rotaviruses with genotypes G1P[8], G2P[4], G3P[8], G4P[8], and G9P[8] (*1*,*2*). Oral live attenuated vaccines were licensed in 2006 in Germany after they were found to be efficient and safe. Monovalent Rotarix (GlaxoSmithKline Biologicals, Rixensart, Belgium) contains a G1P[8] strain, whereas RotaTeq (Merck and Co., West Point, PA, USA) contains 5 bovine-human reassortants representing genotypes G1–4 in association with P[5] and G6P[8] (*3*,*4*). To evaluate rotavirus vaccine efficacy and possible escape of genotypes from host immunity, post-marketing monitoring of circulating wildtype rotaviruses is necessary. That G12, which was detected 1987 in the Philippines, will be a predominant genotype in the future has been assumed. In recent years, a growing number of countries worldwide have reported the occurrence of G12, both sporadically and as a genotype of notable incidence (*5*).

## The Study

A total of 2,752 stool specimens were collected in 2008 from inpatients with diarrhea at Leipzig University Hospital. The samples were derived from 1,804 patients, of whom 715 were <6 years of age. Several aliquots of a 10% stool suspension in phosphate-buffered saline were prepared from each specimen. One aliquot was screened on rotavirus group A antigen by IDEIA (Dako Ltd, Ely, UK). RNA of antigen-positive samples was extracted from a second aliquot by NucliSens easyMAG system (bioMérieux, Boxtel, the Netherlands). Rotavirus gene segments coding for structural viral proteins (VP) 1, VP2, and VP3 were amplified by reverse transcription–PCR with consensus primers (Metabion, Martinsried, Germany): VP1-F 5′-GGCTATTAAAGCTGTACAATG-3′ (nt 1–21), VP1-R 5′-GGTCACATCTAAGCACTC-3′ (nt3302–3285), VP2-F 5′-GGCTATTAAAGGCTCAAT-3′ (nt 1–18), VP2-R 5′-GGTCATATCTCCACAGTGG-3′ (nt 2717–2699), VP3-F 5′-GGCTATTAAAGCAATACTAG-3′ (nt 1–20), VP3-R 5′-GGTCACATCATGACTAGT-3′ (nt 2591–2574), and the other gene segments with primers described elsewhere (*6*–*8*). In the case of gene segments with short untranslated regions, primer ligation and reverse transcription steps were performed as described by Lambden et al. (*9*). Their primer 2 and gene-specific primers were used in subsequent PCRs to determine entire open reading frames.

Amplicons were gel purified by using Wizard SV Gel and PCR Clean-Up System (Promega, Mannheim, Germany) and sequenced by PCR primers and internal primers with the BigDye Terminator v1.1 Cycle Sequencing kit (Applied Biosystems, Foster City, CA, USA) on an ABI Prism 310 Genetic Analyzer (Applied Biosystems). To separate amplicons of mixed infections, the amplicons were cloned into pCRII-TOPO vector and transformed into *Escherichia coli* (Invitrogen, Carlsbad CA, USA). Plasmids were purified by QIAprep Spin Miniprep Kit (QIAGEN, Hilden, Germany) and sequenced with M13 primers (Invitrogen). Corrected chromatograms were assembled by using ContigExpress Module of VectorNTI Suite (Invitrogen). Full-length amino acid sequences were aligned by AlignX (a module of VectorNTI Suite). Phylogenetic analyses were conducted by MEGA version 4.0 software (www.megasoftware.net). Genetic distances were calculated by using the Poisson correction parameter. The dendrograms were constructed by the neighbor-joining method. Statistical support was assessed by bootstrapping with 1,000 replicates (*10*). The sequences of the 2 German G12 rotavirus strains were deposited in GenBank (Table).

**Table:** GenBank accession numbers of both G12 strains of rotavirus found in Germany*

Gene segment	GER126-08†	GER172-08
VP1	FJ747613	FJ747625
VP2	FJ747614	FJ747626
VP3	FJ747615	FJ747627
VP4	FJ747616	FJ747628
VP6	FJ747617	FJ747629
VP7	FJ747618, FJ747619	FJ747630
NSP1	FJ747620	FJ747631
NSP2	FJ747621	FJ747632
NSP3	FJ747622	FJ747633
NSP4	FJ747623	FJ747634
NSP5/6	FJ747624	FJ747635

*VP, viral protein; NSP, nonstructural protein. †Sequences of both G12 and G3 genotypes were present in strain GER126-08.

Of samples from 1,804 patients, 198 (11%) were positive for rotavirus; of those with positive samples, 174 patients were <6 years of age. Genotyping showed G1P[8], G2P[4], G3P[8], G4P[8], G9P[8], G12P[6], G1G9P[8], and G3G12P[8]. G12 rotavirus strains were detected in 3 stool specimens. Strain GER126–08 was derived from the specimen of a 10-year-old boy who had been admitted to the hospital on April 14 because of a first manifestation of type 1 diabetes. After 1 week, he was transferred to our pediatric ward where diarrhea and vomiting developed 3 days later. Rotavirus genotypes of concurrently hospitalized children on the same ward were distinct. G12 strain GER172–08 was found in samples of 2 bottle-fed young infants who had gastroenteritis: a 15-day-old boy on July 31 and a 30-day-old girl on August 11. There was no direct contact between the 3 patients, and none of the patients or their close family members had any migration background or recent travel abroad. They also had not been previously vaccinated against rotavirus.

**Figure 1:**
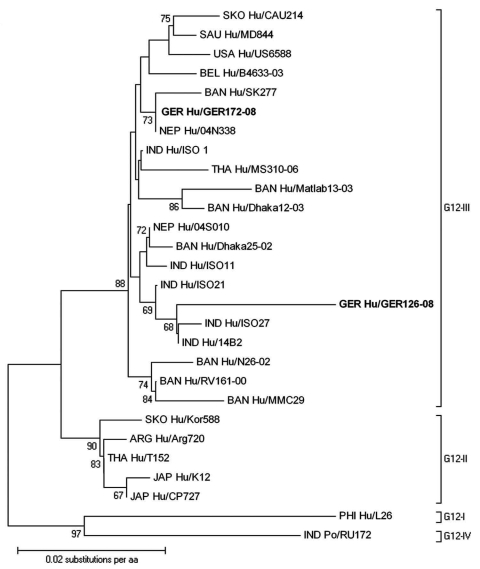
Phylogenetic dendrogram of viral protein 7 (VP7) of G12 rotavirus at the amino acid level. Bootstraps values (1,000 replicates) >65% are shown. The strain name is prefixed by the country of origin (ARG, Argentina; BAN, Bangladesh; BEL, Belgium; GER, Germany; IND, India; JAP, Japan; NEP, Nepal; PHI, Philippines; SAU, Saudi Arabia; SKO, South Korea; THA, Thailand; USA, United States of America) as well as the viral host (Hu, human, Po, porcine). **Boldface** indicates strains of this study. GenBank accession numbers of VP7 genes compared: 04N338 BAF64828, 04S010 BAF64826, 14B2 AAZ79294, Arg720 ACA96827, B4633–03 ABA34217, CAU 214 ABK62858, CP727 BAD24105, Dhaka12–03 ABA34219, Dhaka25–02 ABA34218, ISO1 AAP03062, ISO11 AAY85305, ISO21 AAZ17431, ISO27 AAZ17433, K12 BAD89095, Kor588 ACA96829, L26 ABV53272, Matlab13–03 ABA34220, MD844 BAF02906, MMC29 ACJ54792, MS310–0 BAG83242, N26–02 ABA34221, RU172 ABB17172, RV161–00 ABF67557, SK277 ACJ54800, T152 BAB88671, US6588 ACJ66743.

**Figure 2:**
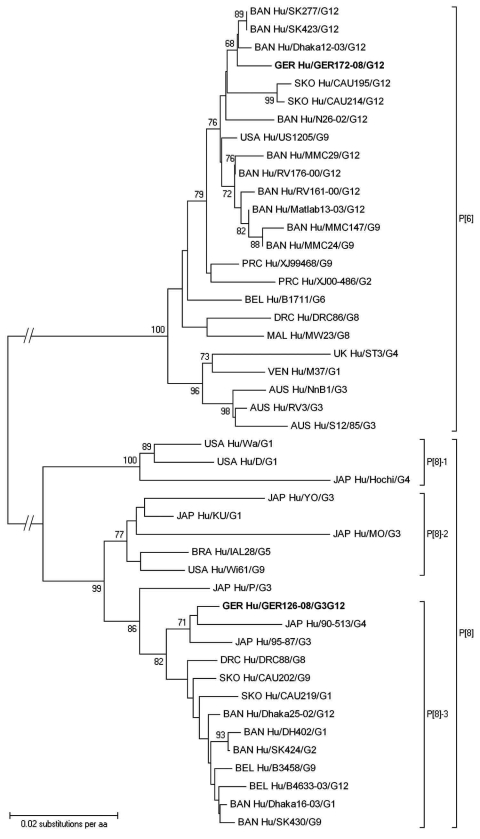
Phylogenetic dendrogram of viral protein 4 (VP4) P[6] and P[8] rotaviruses at the amino acid level. Bootstraps values (1,000 replicates) >65% are shown. The strain name is prefixed by the country of origin (AUS, Australia; BRA, Brazil; BAN, Bangladesh; BEL, Belgium; DRC, Democratic Republic of Congo; GER, Germany; JAP, Japan; MAL, Malawi; PRC, People’s Republic of China; SKO, South Korea; UK, United Kingdom; USA, United States of America; VEN, Venezuela) as well as the viral host (Hu, human) and followed by the associated G genotype. **Boldface** indicates strains of this study. GenBank accession numbers of VP4 genes compared: P[6] B1711 ABU49763, CAU195 ABK62863, CAU214 ABK62864, Dhaka12–03 ABA34207, DRC86 AAY55972, M37 AAA57560, Matlab13–03 ABA34208, MMC147 ACJ54810, MMC24 ACJ54809, MMC29 ACJ54804, MW23 CAB92920, N26–02 ABA34209, NnB1 AAC68884, RV161–00 ABF67555, RV176–00 ABF67561, RV3 AAB05652, S12/85 AAC68883, SK277 ACJ54805, SK423 ACJ54803, ST3 ABV53292, US1205 AAC28852, XJ00–486 ABC49694, XJ99–468 ABC49698; P[8] 90–513 BAF80182, 95–87 BAA77555, B3458 ABV66093, B4633–03 ABA34205, CAU202 ABK62865, CAU219 ACD50869, D ABV53244, DH402 ACJ54815, Dhaka16–03 ABF50136, Dhaka25–02 ABA34206, DRC88 AAY55961, Hochi BAB32852, IAL28 ABV53260, KU BAE76023, MO BAA77543, P ABV53276, SK424 ACJ54811, SK430 ACJ54817, Wa AAA47290, Wi61 ABV53300, YO BAA77544.

Direct sequencing of gel-purified GER126-08 VP7 amplicons resulted in peak superpositions in sequencing gels; the sequencing of clones of this isolate showed a mixture of G3 and G12 genotypes. Contamination was excluded by a second RNA extraction and by comparing sequences to all G3 and G12 types detected in 2008, which were distinct. An amino acid alignment of VP7 G3 showed highest identity (97.5%) to G3 strains from Southeast Asia (data not shown). The G12 genotype belonged to G12-III lineage but was distinct from published full-length sequences, including the only European sequence from Belgium (Figure 1). Highest homology (97.8%) was shown in comparison to the Indian strain 14B2 (Appendix Table). Partial sequence data from European isolates showed no closer relationship, and G12 was not detected in 19 porcine rotaviruses from different piggeries of Saxony (data not shown). Amplification of VP1–4, VP6, and NSP1–5 by gene segment-specific consensus primers within conserved regions in the respective 5′ and 3′ ends was performed. No peak superposition occurred in sequencing of these amplicons, that is, only 1 variant of each genomic RNA segment could be detected. This finding indicates recent reassortment events. Although less likely, minor species of these 10 genomic RNA segments are not entirely excluded; they might have been missed in amplification or cloning and sequencing. The deduced amino acid sequences (Appendix Table) signified a Wa-like genogroup virus (G3G12-P[8]-I1-R1-C1-M1-A1-N1-T1-E1-H1) (*11*,*12*). Its VP4 genomic RNA segment was phylogenetically related to those of Japanese G3 and G4 genotypes (Figure 2) and distinct from all other P[8] genotypes of this collection (data not shown). The origin of the associated genomic RNA segments from either a G3 or a G12 type rotavirus remains unclear, due to the lack of substantial numbers of available full-length sequences.

Rotavirus sequences in stool samples of the 2 newborns were identical. Full-length VP7 amino acid alignments of GER172-08 with published sequences showed 100% homology to G12-III strains ISO16 and ISO29 from India and to strains 04N245, 04N338, 05K021, 05K046, 05K066, and 05N138 from Nepal. The P[6] genotype of GER172-08 was not shared by other rotaviruses in this study, and it showed the highest homology to strains from Bangladesh and South Korea (Figure 2). Analysis of deduced amino acid sequences of all proteins showed a Wa-like genogroup affiliation (G12-P[6]-I1-R1-C1-M1-A1-N1-T1-E1-H1) (data not shown) (*11*,*12*). In a comparative analysis, GER126-08 is clearly distinct from GER172-08 (Appendix Table).

## Conclusions

Two distinct G12 rotaviruses with different P type associations were detected. The findings suggest that they were individually introduced into the local rotavirus diversity. Although GER172-08 is closely related to Southeast Asian strains, the origin of GER126-08 remains unclear. No conclusively related G12 sequence was published or detected in local piggeries.

A G3 VP7 sequence was found in addition in the stool sample GER126-08, however. Mixed infections are fairly common in crowded areas where population density is high and diverse rotavirus strains are co-circulating. They are required for reassortment, the major mechanism of rotavirus evolution (*13*,*14*). Pediatric wards match this setting during rotavirus seasons, because children with different rotavirus strains may be hospitalized simultaneously. Successive nosocomial rotavirus infections during hospitalization may facilitate asynchronous infections that favor reassortment (*15*). The case of the 10-year-old boy in this study fits into this pattern. Indications for recent reassortment events of strain GER126-08 have been detected consistently.

The detection of G12 rotavirus strains in Germany accentuates the need for extended multicenter studies to describe rotavirus diversity and control vaccine efficacy. Rotaviruses in animals should also be included to evaluate the origin of emerging genotypes.

## Supplementary Material

Appendix Table Amino acid identity rates of all genomic RNA segments between rotavirus reference strains and GER126-08*
