# Supplementary material for: Human Infection with G12 Rotaviruses, Germany
Source: Emerg Infect Dis. 2009 Sep;15(9):1512–5. doi: 10.3201/eid1509.090497 (PMC2819891; doi:10.3201/eid1509.090497)
Supplement: Appendix Table — Amino acid identity rates of all genomic RNA segments between rotavirus reference strains and GER126-08* [file 09-0497_appT-s1.pdf]

Appendix Table. Amino acid identity rates of all genomic RNA segments between rotavirus reference strains and GER126-08*

Strain	Genogroup†	Genotype	VP1	VP2	VP3	VP4	VP6	VP7	NSP1	NSP2	NSP3	NSP4	NSP5	NSP6
Wa	Wa-like	G1P[8]	**99.1**	96.8	96.0	94.4	98.2	77.6	82.7	95.8	98.0	96.5	94.4	89.1
DS-1	DS-1-like	G2P[4]	90.1	89.3	80.8	91.3	92.6	75.4	69.3	88.0	82.2	84.0	81.8	80.4
AU-1	AU-1-like	G3P[9]	91.5	91.3	83.2	65.1	92.4	79.1	58.0	88.0	84.1	85.1	87.8	NC‡
P	Wa-like	G3P[8]	98.6	98.4	97.7	97.5	99.4	79.4	83.3	96.2	**99.3**	**97.1**	98.9	NC‡
ST3	Wa-like	G4P[6]	98.0	97.9	96.5	76.9	**99.7**	73.3	92.5	94.6	99.0	93.1	97.9	94.5
B3458	Wa-like	G9P[8]	98.6	97.9	98.4	98.1	99.2	80.9	94.8	94.9	98.3	95.4	**100**	93.4
14B2		G12	NA§	NA§	NA§	NA§	NA§	**97.8**	NA§	NA§	NA§	NA§	NA§	NA§
B4633-03	Wa-like	G12P[8]	98.5	97.2	99.1	98.0	**99.7**	96.9	94.7	99.3	99.0	94.2	**100**	94.5
Dhaka25-02	Wa-like	G12P[8]	98.4	97.2	**99.2**	**98.5**	99.4	96.9	**96.3**	**99.6**	97.4	94.8	**100**	**96.7**
GER172-08	Wa-like	G12P[6]	98.4	**98.5**	99.0	78.3	99.2	96.9	95.5	98.4	98.7	94.8	**100**	93.4
L26	DS-1-like	G12P[4]	90.0	89.2	95.3	91.0	92.1	92.6	68.7	95.2	82.9	84.5	97.9	95.6
Matlab13-03	Wa-like	G12P[6]	98.4	97.1	96.6	78.1	99.4	95.3	**96.3**	99.0	83.2	94.2	**100**	94.5
RU172		G12P[7]	NA§	NA§	NA§	NA§	94.4	92.3	NA§	NA§	NA§	94.8	97.9	**96.7**
RV161-00	DS-1-like	G12P[6]	90.3	89.0	81.0	78.4	92.6	96.6	69.3	89.2	83.5	94.2	82.0	78.2
T152	AU-1-like	G12P[9]	91.3	91.6	84.1	63.8	99.4	95.7	51.9	89.2	83.8	85.7	88.3	78.2

*VP, viral protein; NSP, nonstructural protein; NA, not available. All values given in percentages.
†According to Matthijnssens et al. (*11*), the genogroup was assigned if at least 7 gene segments belonged to the respective Wa-, DS-1-, or AU-1-like genotype. Results of the second G12 strain detected in this study (GER172-08) are highlighted in gray. **Boldface** indicates highest percentages per column. GenBank accession numbers of these highly homologous genomic RNA segments used in comparison: 14B2 VP7 AAZ79294; B3458 NSP5 ABV57763; B4633-03 VP6 ABA34210, NSP5 ABA34247; Dhaka 25-02 VP3 ABA34200, VP4 ABA34206, NSP1 ABA34223, NSP2 ABA34228, NSP6 DQ146659; Matlab13-03 NSP1 ABA34225, NSP5 ABA34250; P NSP3 ABV53278, NSP4 ABV53281; RU172 NSP6 DQ204739 ST3 VP6 ABU87857; Wa VP1 ABF67546.
‡Amino acid identities were not calculated because different open reading frames result in longer and shorter NSP6 proteins, respectively.
§Full-length sequence information was not available.
